# ZW Sex Chromosomes in Australian Dragon Lizards (Agamidae) Originated from a Combination of Duplication and Translocation in the Nucleolar Organising Region

**DOI:** 10.3390/genes10110861

**Published:** 2019-10-30

**Authors:** Kazumi Matsubara, Denis O’Meally, Stephen D. Sarre, Arthur Georges, Kornsorn Srikulnath, Tariq Ezaz

**Affiliations:** 1 Institute for Applied Ecology, Faculty of Science and Technology, University of Canberra, Canberra ACT 2617, Australia; 2 Department of Genetics, Faculty of Science, Kasetsart University, Bangkok 10900, Thailand

**Keywords:** chromosome rearrangements, NOR, evolution, BAC, gene mapping, FISH (fluorescence in situ hybridisation), comparative genomics

## Abstract

Sex chromosomes in some reptiles share synteny with distantly related amniotes in regions orthologous to squamate chromosome 2. The latter finding suggests that chromosome 2 was formerly part of a larger ancestral (amniote) super-sex chromosome and raises questions about how sex chromosomes are formed and modified in reptiles. Australian dragon lizards (Agamidae) are emerging as an excellent model for studying these processes. In particular, they exhibit both genotypic (GSD) and temperature-dependent (TSD) sex determination, show evidence of transitions between the two modes and have evolved non-homologous ZW sex microchromosomes even within the same evolutionary lineage. They therefore represent an excellent group to probe further the idea of a shared ancestral super-sex chromosome and to investigate mechanisms for transition between different sex chromosome forms. Here, we compare sex chromosome homology among eight dragon lizard species from five genera to identify key cytological differences and the mechanisms that may be driving sex chromosome evolution in this group. We performed fluorescence in situ hybridisation to physically map bacterial artificial chromosome (BAC) clones from the bearded dragon, *Pogona vitticeps’* ZW sex chromosomes and a nucleolar organising region (NOR) probe in males and females of eight Agamid species exhibiting either GSD or TSD. We show that the sex chromosome derived BAC clone hybridises near the telomere of chromosome 2q in all eight species examined. This clone also hybridises to the sex microchromosomes of three species (*P vitticeps*, *P. barbata* and *Diporiphora nobbi*) and a pair of microchromosomes in three others (*Ctenophorus pictus*, *Amphibolurus norrisi* and *Amphibolurus muricatus*). No other chromosomes are marked by the probe in two species from the closely related genus *Physignathus*. A probe bearing nucleolar organising region (NOR) sequences maps close to the telomere of chromosome 2q in all eight species, and to the ZW pair in *P. vitticeps* and *P. barbata*, the W microchromosome in *D. nobbi*, and several microchromosomes in *P. cocincinus*. Our findings provide evidence of sequence homology between chromosome 2 and the sex chromosomes of multiple agamids. These data support the hypothesis that there was an ancestral sex chromosome in amniotes that gave rise to squamate chromosome 2 and raises the prospect that some particular property of this chromosome has favoured its role as a sex chromosome in amniotes. It is likely that the amplification of repetitive sequences associated with this region has driven the high level of heterochromatinisation of the sex-specific chromosomes in three species of agamid. Our data suggest a possible mechanism for chromosome rearrangement, including inversion and duplication near the telomeric regions of the ancestral chromosome 2 and subsequent translocation to the ZW sex microchromosomes in three agamid species. It is plausible that these chromosome rearrangements involving sex chromosomes also drove speciation in this group.

## 1. Introduction

Sex chromosomes are a common feature of organisms with genotypic sex determination (GSD) and generally take the form XX/XY (male heterogamety) or ZZ/ZW (female heterogamety). The former is typical of mammals but is also found in reptiles and fish, while the latter are found in birds and frequently in reptiles [1]. Sex chromosomes have been implicated in speciation events through the generation of post zygotic incompatibility among divergent taxa, including taxa with either nascent or advanced XY and ZW sex chromosome systems [2,3,4,5,6,7,8]. The morphology and gene content of sex chromosomes are highly variable among amniotes and often differ between closely related species [1,9,10,11]. Genome sequence analyses and cross-species gene mapping have established that the synteny seen among sex chromosome genes in some reptiles is shared with distantly related amniotes and often involves genomic regions orthologous to squamate chromosome 2 [12]. This finding led us to hypothesise that the ancestral squamate chromosome 2 was part of a larger ancestral amniote super-sex chromosome containing large segments whose synteny is conserved among extant amniotes and that multiple chromosomal rearrangements have occurred during the evolution of sauropsid sex chromosomes [12].

The Australian dragon lizards (Agamidae) provide an excellent model for understanding sex chromosome evolution because of their diversity of sex determining mechanisms, multiple transitions between GSD and TSD (temperature-dependent sex determination), and novel sex chromosomes [10,13,14,15,16,17]. By definition, TSD species lack sex chromosomes. Karyotypes of many TSD species are indistinguishable from their GSD relatives that possess cryptic sex chromosomes [9,10,11,13]. This suggests that very little change, whether it be at the genetic or epigenetic level, is required for transition between modes [14,17,18,19]. Sister taxa that exhibit different sex determination modes and species in which sex reversal of individuals with heteromorphic sex chromosomes can be induced through high incubation temperature [14,17] are of particular interest. The central bearded dragon (*Pogona vitticeps*), exhibits a ZZ/ZW sex chromosome system where the Z chromosome is homologous with chicken (*Gallus gallus*) chromosome 17 and 23 and the W chromosome is highly heterochromatic [9,10,11,16,20,21]. Dense gene maps for *P. vitticeps* show that the sex chromosome pair and the terminal region adjacent to the nucleolar organising region (NOR) on chromosome 2 share homology, suggesting duplication, translocation or fission–fusion [16,20,21]. These rearrangements (e.g., inversion, duplications and translocation) provide evidence that the bearded dragon ZW pair once shared synteny with squamate chromosome 2, suggesting that rearrangements involving the NOR on chromosome 2 have been involved in the formation of sex microchromosomes [12].

To better understand the evolution of agamid sex chromosomes and those of *P. vitticeps* specifically, we have developed a number of molecular resources for comparative genomic analyses that include a sex chromosome ‘paint’ [13], sex-linked markers [14,15] and a bacterial artificial chromosome (BAC) anchored physical map [16,20,21]. Previously, we reported evidence for chromosome fusions involving two pairs of microchromosomes and the W chromosome of *P. vitticeps* and an association between the NOR bearing telomeric region on chromosome 2q and the sex chromosomes in *P. vitticeps* [16,22]—an observation that has been reported in many taxa, from fish to eutherian mammals [23]. Thus, it appears that the sex chromosomes of at least some dragon lizards share homology with sex-related elements of other taxa, despite their apparent diversity of sex determining mechanisms. Here, we use the NOR and *P. vitticeps* sex chromosome-derived probes to perform comparative chromosome mapping in an additional eight representative species of GSD and TSD dragon lizards. All but two species in this study have a diploid chromosome number of 2n = 32 (12 macro- and 20 microchromosomes), while two species of *Physignathus* (*P. lesueurii* and *P. cocincinus*) have 2n = 36 (12 macro- and 24 microchromosomes) with two additional pairs of microchromosomes [24,25]. We selected these eight species for comparison based on their modes of sex determination. Three species (*Pogona vitticeps*, *P. barbata* and *Diporiphora nobbi*), have GSD with ZW sex microchromosomes; two species (*Amphibolurus norrisi* and *Ctenophorus pictus*) have GSD with cryptic sex chromosomes, two species (*Amphibolurus muricatus* and *Physignathus lesueurii*) have TSD and we included one species (*Physignathus cocincinus*) whose sex determination mode is unknown, as shown in Table 1. We use comparative mapping of these probes in combination with our previously published data to delineate the process of chromosomal rearrangement and discuss the implications of our findings for the origin of sex chromosomes in Australian dragon lizards.

## 2. Materials and Methods

### 2.1. Animals, Sexing, Cell Culture and Chromosome Preparations

We examined eight dragon lizard species (seven Australian species and one Asian species as an outgroup) representing five genera, as shown in Table 1. 

Animals were euthanised by an intraperitoneal injection of sodium pentobarbitone at a concentration of 150 mg/Kg body weight. Phenotypic sex was determined through external morphology, hemipenes eversion [26] and by internal examination of gross gonadal morphology [9]. Metaphase chromosomes were prepared either from short-term culture of whole blood or from fibroblast cell lines as described by Ezaz et al. [13]. Chromosome slides were treated with 100 µg/mL RNase for 1 h at 37 °C and then rinsed three times in 2X sodium saline citrate (SSC) before storage at −80 °C until use. Animal care and experimental procedures were performed following the guidelines of the Australian Capital Territory Animal Welfare Act 1992 (Section 40) and conducted under approval of the Committee for Ethics in Animal Experimentation at the University of Canberra (Permit Number: CEAE 11/07).

### 2.2. Probe Preparation and Fluorescence in Situ Hybridisation (FISH)

To identify regions orthologous to *P. vitticeps* sex chromosomes, we used the BAC clone Pv151P16 (referred to hereafter as PviZW BAC) characterised by Ezaz et al. [20] in cross-species mapping experiments. To map the NOR, we used a probe derived from a tammar wallaby (*Macropus eugenii*) BAC clone (AGI329J14) bearing 18S and 28S rDNA genes [16,27]. We used a wallaby-derived probe because we found the absence of species-specific NOR-associated repeats gave consistently cleaner hybridisation signals in our study species. While this NOR probe hybridises on to both active and inactive sites [16], we use it here as a marker for physical mapping. BAC DNA was extracted using the Promega Wizard Plus SV Minipreps DNA Purification System (Promega, Madison, WI, USA) following the manufacturer’s protocol with volumes scaled up for 15 mL cultures. Cross-species FISH mapping was performed with 200–500 ng of BAC DNA labelled by nick translation, incorporating either Spectrum Orange- or Spectrum Green-conjugated dUTP (Abbott Molecular), for 2 h at 15 °C [27]. The labelled probes were precipitated and resuspended in hybridisation buffer and approximately 12–15 µL dropped on to a glass slide containing metaphases and hybridised overnight to 24 h at 37 °C. The slides were washed once in 0.4X SSC and 0.3% IGEPAL (Sigma-Aldrich) at 60 °C for 2–3 min, then once in 2X SSC and 0.1% IGEPAL at room temperature for 1–2 min [9], dehydrated through an ethanol series (1 min in 70%, 90% and 100% ethanol), air dried, stained with 50 µg/mL DAPI (4′, 6′-diamidino-2-phenylindole) in 2 × SSC for 30–45 s at room temperature and finally mounted with Vectashield (Vector Laboratories, Burlingame, CA, USA). Images were captured using a Zeiss Axio Scope A1 epifluorescence microscope fitted to a high-resolution microscopy camera AxioCam MRm Rev. 3 (Carl Zeiss Australia Pty Ltd., Sydney, Australia) and analysed using the ISIS Fluorescence Imaging System (MetaSystems) (Carl Zeiss Australia Pty Ltd, Sydney, Australia). 

### 2.3. BLASTn Analysis of PviZW BAC (Pv151P16) 

We queried the chicken and green anole genomes with PviZW BAC sequences using the default parameters of BLASTn [28] to identify orthologies, in particular, with chicken sex chromosomes and with anole chromosomes 2.

## 3. Results

### 3.1. Physical Mapping of PviZW BAC

**Figure 1:**
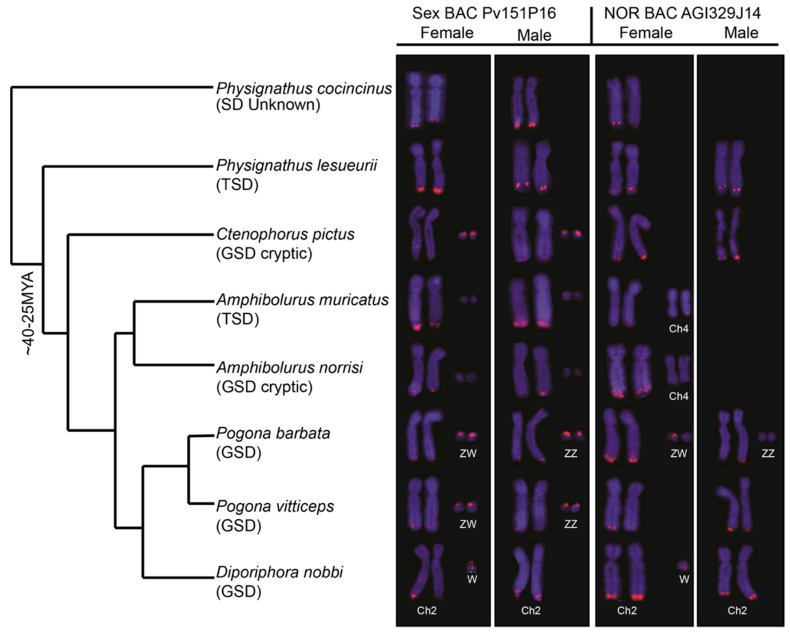
Physical mapping of *Pogona vitticeps* sex chromosome BAC (bacterial artificial chromosome) clone Pv151P16 (PviZW) and nucleolar organising region (NOR) (18S–28S rDNA) containing BAC clone AGI329J14 in eight species of dragon lizards showing locations and variable intensities of hybridisation signals on chromosome 2, ZW sex chromosomes, a pair of microchromosomes and a pair of chromosome 4. Both BAC clones were hybridised onto the telomeric region of chromosome pair 2 and ZW sex chromosomes in *P. barbata*. The PviZW BAC clone hybridised onto the telomeric region of chromosome pair 2 and the ZW sex chromosomes in *P. vitticeps*, while the NOR BAC clone hybridised onto the telomeric region of chromosome pair 2 only and not onto the ZW sex chromosomes or on any other chromosomes. This pattern of hybridisation was also observed in two other GSD species with cryptic sex chromosomes and one TSD species. We were unable to determine whether the microchromosome pair were sex chromosomes in those species. Similar patterns were also observed in *D. nobbi*, except that no hybridisation signal was observed on the Z chromosome for either probes. Hybridisation signal from the NOR BAC clone onto chromosome 4 was observed in *A. muricatus* and *A. norrisi*. The truncated phylogeny (not according to scale) is derived from [29]. Chromosomal locations of BACs in *P. vitticeps* were obtained from [16].

As has previously been observed [23], the PviZW BAC mapped to the Z and W chromosomes in *P. vitticeps* with an intense signal on the W chromosome compared with that on the Z, as shown in Figure 1. Faint hybridisation signals were also observed adjacent to the telomere of the long arm of chromosome 2 (2qter). A similar hybridisation pattern (Z, W, chromosome 2) was observed in *P. barbata*, while in *D. nobbi*, only chromosome pair 2 and the Z chromosome (but not the W chromosome) were marked by the probe. Hybridisation patterns in the two *Amphibolurus* species and *C. pictus* were similar to those in *P. vitticeps*, *P. barbata* and *D. nobbi*, where PviZW BAC mapped to a pair of microchromosomes and chromosome 2qter. Whether these microchromosomes are sex chromosomes in *Ctenophorus fordi* and the *Amphibolurus* species was not determined. The fluorescence hybridisation signals on microchromosomes were faint in *Amphibolurus* compared with those in the three remaining genera. However, signals on chromosome 2qter of *Amphibolurus* were at a higher intensity, similar to those of *Physignathus*, than those in the other three genera. Apart from chromosome 2qter, no pair of chromosomes was consistently marked by hybridisation signals in the two species of *Physignathus*, as shown in Figure 1.

### 3.2. Physical Mapping of 18S–28S rDNA (NOR) Genes

**Figure 2:**
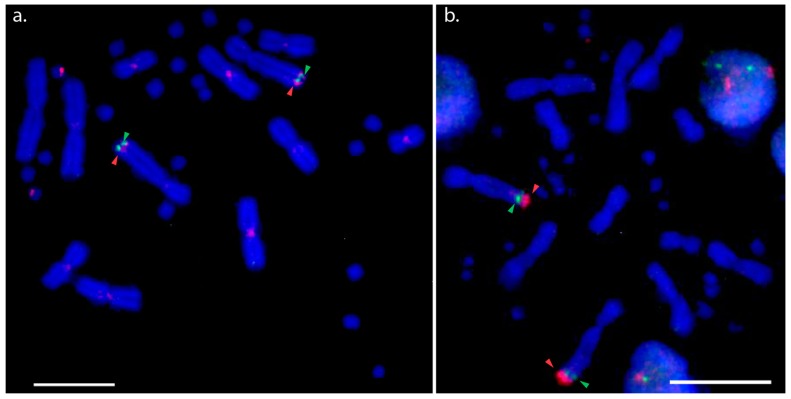
Two-colour FISH (fluorescence in situ hybridisation) with BAC clones Pv151P16 (PviZW) and AGI329J14 (NOR) in *Pogona vitticeps* and *Physignathus lesueurii*. (**a**) Two-colour FISH showing chromosomal locations of *P. vitticeps* (GSD species) sex chromosome BAC clone PviZW (red hybridisation signals) and NOR BAC clone AGI329J14 (green hybridisation signals) on chromosome 2, Z and W chromosomes in *P. vitticeps*; (**b**) two-colour FISH showing chromosomal locations of *P. vitticeps* sex chromosome BAC clone PviZW (red hybridisation signals) and NOR BAC clone AGI 329J14 (green hybridisation signals) on chromosome 2 in *Physignathus lesueurii* (TSD species). Red and green arrows indicate locations of these two BAC clones in both species. The colocation of the red signal is proximal to the green signal in GSD species *P. vitticeps* (**a**), while the red signal is distal to the green signal in TSD species *Physignathus lesueurii*, implying an inversion event involving NOR bearing BAC clone and PviZW BAC clone in these two species. Note that in these experiments, FISH was performed without any suppressor DNA, therefore, some centromeric signals are visible on several macrochromosomes of *P. vitticeps* (**a**) which are due to the high repeat content of the PviZW BAC clone [20]. Scale bar represents 10 μm.

Hybridisation of the NOR probe (BAC AGI329J14) was observed in the distal region near the telomere of the long arm of chromosome 2q for all species examined, except for the two *Physignathus* species and *A. muricatus* where the location on 2q tended to be more proximal, as shown in Figure 1. The hybridisation signals of the NOR probe were similar to the PviZW BAC for both *D. nobbi* and *P. barbata*. Comparatively bright hybridisation signals were observed on the Z sex microchromosome and there were faint signals on the W sex microchromosome in *P. barbata*. In *D. nobbi*, we observed weaker signals on the W sex microchromosome in addition to signals on chromosome 2qter. The NOR probe also mapped to chromosome 4qter in both species of *Amphibolurus* and to multiple microchromosomes in *P. cocincinus*, as shown in Figure S1. In addition to single colour FISH, two-colour FISH of PviZW BAC and the NOR probe was performed on *P. vitticeps* and *P. lesueurii* to resolve the location of the NOR with respect to PviZW BAC. In these cases, the PviZW BAC mapped distally to the NOR, adjacent to the telomere in *P. vitticeps* and proximally with respect to the NOR in *P. lesueurii*, as shown in Figure 2.

### 3.3. Blast Analysis of PviZW BAC

Blast analysis of the PviZW BAC clone (Pv151P16) against the chicken genome revealed sequence homologies with multiple chicken chromosomes including chicken chromosome Z and W, as shown in Table S1, while blast analysis of this BAC clone against the green anole genome (*Anolis carolinensis*) revealed homologies with the green anole chromosome 2, as shown in Table S2.

## 4. Discussion

The diversity of sex chromosomes among amniotes is epitomised by the independent evolution of sex chromosomes and sex determining modes and yet sex chromosome sequences are partially shared across several lineages [12]. The squamate reptile chromosome 2 is common to these homologies because syntenic blocks from chromosome 2 share homology with sex chromosomes in other amniotes and it is therefore possible that these blocks represent components of an ancestral super-sex chromosome [12,30]. Here, we confirmed that the sex-linked PviZW BAC maps to the Z and W chromosomes in *P. vitticeps* (as well as *D. nobbi* and *P. barbata*) but we also found that it maps faintly to the telomere of the long arm of chromosome 2 (2qter) and exhibits similar hybridisation patterns across five other dragon lizards from *Diporiphora*, *Amphibolurus*, *Ctenophorus* and *Physignathus.* These findings provide further evidence of sequence homology between chromosome 2 and the sex chromosomes of agamids.

Hybridisation of the same BAC onto two different chromosome pairs, as seen with chromosome 2 and microchromosomes of most of the agamids examined, might result from the presence of common genomic elements such as repetitive sequences [20]. The different intensity in signal among lizards from the mapped PviZW BAC clone suggests that the amplification of the repetitive sequences in that BAC clone has occurred independently in each lineage and at different rates. This is similar to the homology shared between the squamate reptile chromosome 2 and the sex chromosomes of chicken and snake [30,31], and the homology observed between the chicken Z and W chromosomes and chromosome 2 of *Anolis carolinensis*, as shown in Tables S1 and S2.

**Figure 3:**
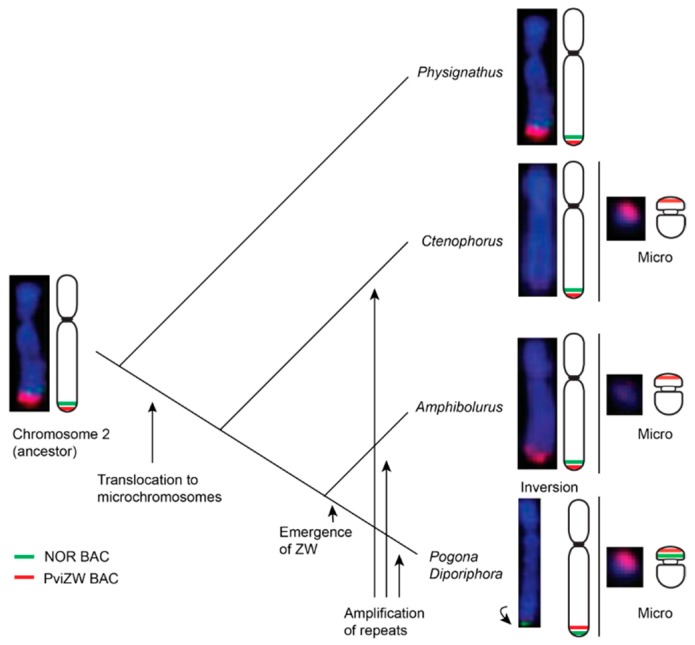
Schematic diagram showing plausible mechanisms of chromosomal rearrangements and evolution of sex chromosomes in GSD dragon lizards. Chromosomal locations of *Pogona vitticeps* sex chromosome BAC clone Pv151P16 and NOR BAC clone AGI329J14 are indicated in red and green colours, respectively. Arrows indicate possible evolutionary events including translocation, amplification of repeats and inversion leading to the evolution of the ZZ/ZW sex microchromosome system in *Pogona* and *Diporiphora* lineages. Truncated phylogeny (not according to scale) is derived from [29].

We also located hybridisation signals from our NOR probe near the telomeres of chromosome 2 in all species, with additional signals on the sex chromosomes of *P. barbata* and *D. nobbi*, on most microchromosomes of *P. cocincinus*, as shown in Figure S1, and on the telomeric region of chromosome 4 in two *Amphibolurus* species. NORs, comprising 18S–28S rDNA genes, have previously been similarly located on a pair of microchromosomes or on chromosome 2 in Iguania [32], but have also been reported on the long arms of chromosome 1 and chromosome 6 in *Leiolepis* and *Tropidurus* spp., respectively [33,34,35]. An association between the NOR and sex chromosomes has been reported in taxa as divergent as fish, amphibians, marsupials and eutherian mammals and it is hypothesised that the NOR plays a role in heterochromatinisation, leading to the suppression of recombination between proto sex chromosomes [23,36,37]. Ribosomal DNA genes may therefore be important in both autosomal and sex chromosomal rearrangements via unequal crossing over and non-homologous recombination in amniotes [34,38]. Our data suggest that the association observed between the NOR and PviZW BAC clones in agamid sex microchromosomes (as well as other microchromosomes) has arisen by duplication of the region adjacent to the NOR near the telomere on chromosome 2 followed by translocation (terminal transposition) either to a pre-existing microchromosome or to create a new microchromosome, as shown in Figure 3. 

**Figure 4:**
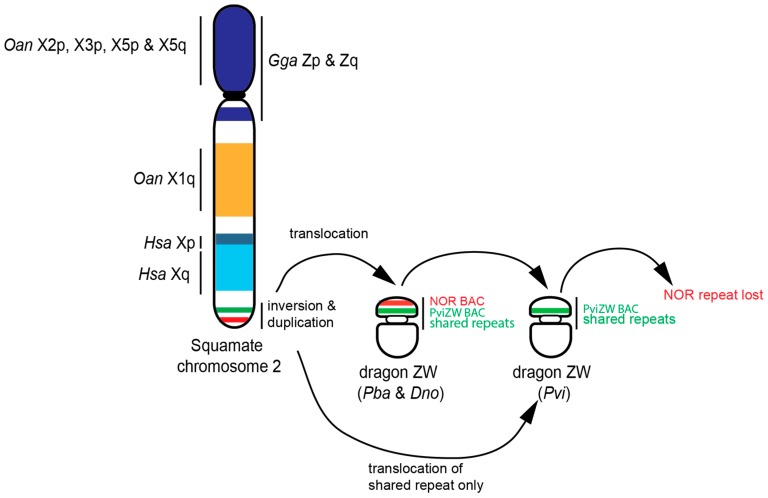
Schematic diagram showing hypothetical model of ZW sex chromosome evolution in Australian dragon lizards, supporting the hypothesis that squamate chromosome 2 is part of ancestral super-sex chromosomes. *Gga*: *Gallus gallus*; *Hsa*: *Homo sapiens*; *Oan*: *Ornithorhynchus anatinus*; *Pba*: *Pogona barbata*, *Pvi*: *Pogona vitticeps*; *Dno*: *Diporiphora nobbi*; X: chromosomes; Z: Z chromosomes; W: W chromosome; p: short arm; q: long arm.

Given that this translocation is present in all lineages other than *Physignathus*, it is likely that the duplication/translocation occurred approximately 21 Mya [29] following the divergence of *Physignathus* but before the divergence of all other Australasian agamids. The absence of NOR-associated sequences on the sex microchromosome in *P. vitticeps*, but their presence on the sex microchromosome in *P. barbata* and *D. nobbi*, implies that this particular region has been lost from *P. vitticeps* following an initial translocation. Alternatively, it is conceivable, although less parsimonious, that the sex microchromosomes of *P. vitticeps* were formed by, or were the recipient of, a separate partial translocation of the region containing PviZW (but not the NOR-associated sequences) from the ancestral chromosome 2, as shown in Figure 4. 

Two-colour FISH mapping reveals the orientation of two probes with respect to the centromere. The regions marked by PviZW and the NOR probe in *P. vitticeps* showed an inverted orientation of hybridisation signals with respect to the regions marked on chromosome 2 in *P. lesueurii*, suggesting that a paracentric inversion has occurred. The PviZW BAC also hybridised to a pair of microchromosomes in two species of *Amphibolurus* (*A. muricatus*; TSD and *A. norrisi*; GSD with cryptic sex chromosomes) and in *C. pictus* (GSD with cryptic sex chromosomes) but we could not confirm that those microchromosomes were also sex chromosomes. Further work is required to test that proposition. The weak hybridisation signal of PviZW BAC on microchromosomes of the *Amphibolurus* species may also indicate an accumulation of repeats divergent between *P. vitticeps’* sex microchromosomes and the microchromosomes in the *Amphibolurus* species. 

The presence of elements from chromosome 2 on the sex chromosomes of agamids is concordant with our physical mapping of these chromosomes [9,10,16,20,21] and suggests that the sex chromosomes of *D. nobbi*, *P. barbata* and *P. vitticeps* share a common origin from the ancestral squamate chromosome 2. In particular, our study suggests that a paracentric inversion followed by a simple translocation gave rise to the ZW sex microchromosomes in two out of three GSD species (e.g., in *P. barbata* and in *D. nobbi*), while the NOR-associated repeat sequences have been lost in the third GSD species, *P. vitticeps*, after its divergence from their common ancestor, as shown in Figure 4. Overall, these data support the hypothesis that there was an ancestral super-sex chromosome in amniotes [12,31] and raises the prospect that some particular property of this chromosome favoured its role as the sex chromosome in amniotes [1,39]. It is also likely that the amplification of repetitive sequences associated with this region has driven the high level of heterochromatinisation seen in the sex-specific chromosomes, particularly W chromosomes in *D. nobbi*, *P. barbata* and *P. vitticeps*. 

## 5. Conclusions

Chromosome rearrangements play a major role in speciation and it is also clear that they are also important in the evolution of sex chromosomes. Here, we have provided evidence that chromosome rearrangements, such as inversions involving the NOR, duplication and translocation, have been significant in the evolution of ZW sex chromosomes in at least three species of Australian dragon lizards, as shown in Figure 3 and Figure 4. Our study also provides support for the hypothesis that squamate chromosome 2 represents an ancestral superchromosome for amniote sex chromosomes. It is plausible that these chromosome rearrangements involving sex chromosomes also drove speciation in this group. Investigations including more dragon lizards and other iguanian lizards are required for a better understanding of sex chromosome evolution as well as sex chromosome driven speciation events in squamate reptiles. 

## Figures and Tables

**Table 1:** Table showing list of species, chromosome numbers, location of origin, modes of sex determination and numbers of individuals used in this study.

Species	Diploidy (2n), SD and SC	Locality and Origin	Number of Animals Used (F + M)	
			Pv151_P16	AGI 329_J14
*Diporiphora nobbi*	32, GSD (ZW)	Vic, Australia	1 + 1	1 + 1
*Pogona vitticeps*	32, GSD (ZW)	NSW, Australia	1 + 1	1 + 1
*Pogona barbata*	32, GSD (ZW)	ACT, Australia	1 + 1	1 + 1
*Amphibolurus norrisi*	32, GSD	Vic, Australia	1 + 1	1 + 0
*Amphibolurus muricatus*	32, TSD	ACT, Australia	1 + 1	1 + 0
*Ctenophorus pictus*	32, GSD	NSW, Australia	1 + 1	1 + 1
*Physignathus lesueurii*	36, TSD	ACT, Australia	1 + 1	1 + 1
*Physignathus cocincinus*	36, NK	Pet trade, Asia	1 + 1	1 + 0

GSD: genotypic sex determination; TSD: temperature-dependent sex determination; NK: not known; SD: sex determination; SC: sex chromosomes; ACT: Australian Capital Territory; NSW: New South Wales; Vic: Victoria; F: female; M: male.

## Supplementary Materials

The following are available online at https://www.mdpi.com/2073-4425/10/11/861/s1, Figure S1: Chromosomal localisations of AGI 329-J14 BAC containing 18S–28S rDNA genes in Physignathus cocincinus. Figure shows intense signals near the telomeric region of chromosome 2 and in most microchromosomes. Scale bar represents 10μm, Table S1: Genome content of chicken (Gallus gallus) homologous to Pogona vitticeps BAC clone Pv151P16 (GenBank accession KF541652.1 https://www.ncbi.nlm.nih.gov/nuccore/KF541652) derived from as identified by BLASTn with the NCBI database (http://blast.ncbi.nlm.nih.gov/Blast.cgi); Gga: Gallus gallus, Table S2: Genome content of green Anole (Anolis carolinensis) homologous to Pogona vitticeps BAC clone Pv151P16 as identified by BLASTn with the NCBI database (http://blast.ncbi.nlm.nih.gov/Blast.cgi); Aca: Anolis carolinensis.
