# Supplementary material for: ZW Sex Chromosomes in Australian Dragon Lizards (Agamidae) Originated from a Combination of Duplication and Translocation in the Nucleolar Organising Region
Source: Genes (Basel). 2019 Oct 30;10(11):861. doi: 10.3390/genes10110861 (PMC6895791; doi:10.3390/genes10110861)
Supplement: Supplementary file 1 [file genes-10-00861-s001.zip › Genes_Matsubara et al_Tables S1 and S2_23 Sep.docx]

**Table S1.** Genome content of chicken (*Gallus gallus*) homologous to *Pogona vitticeps* BAC clone Pv151P16 (GenBank accession KF541652.1 https://www.ncbi.nlm.nih.gov/nuccore/KF541652) derived from as identified by BLASTn with the NCBI database (<http://blast.ncbi.nlm.nih.gov/Blast.cgi>); Gga: *Gallus gallus*.

| **Description** | **Score** | **E-value** | **Accession** | **Gga chromosomal location** |
| --- | --- | --- | --- | --- |
| PREDICTED: Gallus gallus zinc finger protein 184-like (LOC429800), mRNA | 171 | 3.00E-38 | XM_015295103 | 16 |
| Gallus gallus BAC clone J_AA173O01 from chromosome unknown, complete sequence | 171 | 3.00E-38 | AC275299 | unknown |
| Gallus gallus hypoxia inducible factor 1 subunit alpha (HIF1A), mRNA | 148 | 4.00E-31 | NM_204297 | 5 |
| Gallus gallus mRNA for hypoxia-inducible factor-1 alpha, complete cds | 148 | 4.00E-31 | AB013746 | 5 |
| PREDICTED: Gallus gallus hypoxia inducible factor 1 alpha subunit (HIF1A), transcript variant X5, misc_RNA | 146 | 1.00E-30 | XR_001466725 | 5 |
| Gallus gallus finished cDNA, clone ChEST230n16 | 145 | 1.00E-30 | CR390299 | 5 |
| Gallus gallus finished cDNA, clone ChEST933g8 | 145 | 1.00E-30 | CR386431 | 5 |
| PREDICTED: Gallus gallus zinc finger protein 184-like (LOC769660), transcript variant X2, misc_RNA | 144 | 5.00E-30 | XR_003072622 | 33 |
| PREDICTED: Gallus gallus zinc finger protein 184-like (LOC769660), transcript variant X1, mRNA | 144 | 5.00E-30 | XM_015272565 | 33 |
| PREDICTED: Gallus gallus zinc finger protein 7-like (ZNF7L), mRNA | 127 | 4.00E-25 | XM_003643274 | 33 |
| Gallus gallus BAC clone CH261-110N3 from chromosome z, complete sequence | 124 | 5.00E-24 | AC189035 | Z |
| Gallus gallus BAC clone CH261-85A21 from chromosome z, complete sequence | 124 | 5.00E-24 | AC187271 | Z |
| Gallus gallus staufen double-stranded RNA binding protein 1 (STAU1), mRNA | 124 | 5.00E-24 | NM_001012831 | 20 |
| Gallus gallus mRNA for hypothetical protein, clone 32c1 | 124 | 5.00E-24 | AJ721000 | 20 |
| Gallus gallus BAC clone CH261-183A10 from chromosome w, complete sequence | 120 | 6.00E-23 | AC177807 | W |
| Gallus gallus clone TAM31-10L3, complete sequence | 120 | 6.00E-23 | AC138565 | 1 |
| Gallus gallus finished cDNA, clone ChEST447p21 | 120 | 6.00E-23 | CR390483 | 20 |
| Gallus gallus BAC clone CH261-173O18 from chromosome unknown, complete sequence | 120 | 6.00E-23 | AC159212 | 1 |
| Gallus gallus BAC clone TAM31-16O5 from chromosome w, complete sequence | 118 | 2.00E-22 | AC163710 | W |
| Gallus gallus BAC clone CH261-114G22 from chromosome w, complete sequence | 118 | 2.00E-22 | AC182258 | W |
| Gallus gallus BAC clone CH261-119F24 from chromosome unknown, complete sequence | 118 | 2.00E-22 | AC147968 | unknown |
| Gallus gallus finished cDNA, clone ChEST72o14 | 116 | 7.00E-22 | CR354330 | unknown |
| Gallus gallus BAC clone CH261-75N4 from chromosome w, complete sequence | 114 | 2.00E-21 | AC175832 | W |
| Gallus gallus BAC clone CH261-78E22 from chromosome w, complete sequence | 114 | 2.00E-21 | AC182260 | W |
| Gallus gallus BAC clone TAM31-16L19 from chromosome w, complete sequence | 114 | 2.00E-21 | AC182259 | W |
| PREDICTED: Gallus gallus zinc finger protein 606-like (ZNF606L), transcript variant X9, mRNA | 113 | 8.00E-21 | XM_025141662 | 18 |
| PREDICTED: Gallus gallus zinc finger protein 606-like (ZNF606L), transcript variant X8, mRNA | 113 | 8.00E-21 | XM_025141661 | 18 |
| PREDICTED: Gallus gallus zinc finger protein 606-like (ZNF606L), transcript variant X7, mRNA | 113 | 8.00E-21 | XM_025141660 | 18 |
| PREDICTED: Gallus gallus zinc finger protein 606-like (ZNF606L), transcript variant X6, mRNA | 113 | 8.00E-21 | XM_015295210 | 18 |
| PREDICTED: Gallus gallus zinc finger protein 606-like (ZNF606L), transcript variant X5, mRNA | 113 | 8.00E-21 | XM_025141659 | 18 |
| PREDICTED: Gallus gallus zinc finger protein 606-like (ZNF606L), transcript variant X4, mRNA | 113 | 8.00E-21 | XM_015295209 | 18 |
| PREDICTED: Gallus gallus zinc finger protein 606-like (ZNF606L), transcript variant X3, mRNA | 113 | 8.00E-21 | XM_015295208 | 18 |
| PREDICTED: Gallus gallus zinc finger protein 606-like (ZNF606L), transcript variant X2, mRNA | 113 | 8.00E-21 | XM_015295205 | 18 |
| PREDICTED: Gallus gallus zinc finger protein 606-like (ZNF606L), transcript variant X1, mRNA | 113 | 8.00E-21 | XM_015295199 | 18 |
| Gallus gallus BAC clone CH261-191E1 from chromosome z, complete sequence | 112 | 3.00E-20 | AC202713 | Z |
| Gallus gallus BAC clone CH261-152H24 from chromosome z, complete sequence | 112 | 3.00E-20 | AC187588 | Z |
| Gallus gallus BAC clone CH261-170C22 from chromosome z, complete sequence | 111 | 3.00E-20 | AC202717 | Z |
| Gallus gallus BAC clone CH261-122M13 from chromosome z, complete sequence | 111 | 3.00E-20 | AC188446 | Z |
| Gallus gallus BAC clone CH261-96E14 from chromosome z, complete sequence | 110 | 1.00E-19 | AC216792 | Z |
| Gallus gallus BAC clone CH261-166F20 from chromosome z, complete sequence | 109 | 1.00E-19 | AC187268 | Z |
| Chicken DNA sequence from clone WAG-4C11, complete sequence | 109 | 1.00E-19 | BX663523 | unknown |
| Gallus gallus BAC clone CH261-146A23 from chromosome z, complete sequence | 108 | 3.00E-19 | AC197509 | Z |
| Gallus gallus BAC clone CH261-127N1 from chromosome z, complete sequence | 108 | 3.00E-19 | AC189110 | Z |
| Gallus gallus clone CW nonLTR retrotransposon CR1, partial sequence | 107 | 3.00E-19 | AF308606 | unknown |
| Gallus gallus fosmid J_AE-44E9, complete sequence | 106 | 1.00E-18 | AC270448 | W |
| Gallus gallus fosmid J_AE-13G13, complete sequence | 106 | 1.00E-18 | AC270317 | W |
| Gallus gallus BAC clone TAM32-10H12 from chromosome z, complete sequence | 106 | 1.00E-18 | AC201860 | Z |
| Gallus gallus BAC clone CH261-158M5 from chromosome z, complete sequence | 106 | 1.00E-18 | AC188580 | Z |
| Gallus gallus BAC clone CH261-64N18 from chromosome z, complete sequence | 106 | 1.00E-18 | AC188945 | Z |
| Gallus gallus BAC clone CH261-15A16 from chromosome z, complete sequence | 106 | 1.00E-18 | AC188808 | Z |
| Gallus gallus BAC clone CH261-28G3 from chromosome z, complete sequence | 106 | 1.00E-18 | AC189680 | Z |
| Gallus gallus BAC clone CH261-175O22 from chromosome z, complete sequence | 106 | 1.00E-18 | AC188693 | Z |
| Gallus gallus BAC clone CH261-39D10 from chromosome z, complete sequence | 106 | 1.00E-18 | AC187364 | Z |
| Gallus gallus BAC clone CH261-93P21 from chromosome z, complete sequence | 104 | 4.00E-18 | AC188815 | Z |
| Chicken DNA sequence from clone WAG-93H17, complete sequence | 104 | 4.00E-18 | BX663526 | unknown |
| Gallus gallus BAC clone CH261-4H14 from chromosome unknown, complete sequence | 104 | 4.00E-18 | AC145975 | unknown |
| Gallus gallus BAC clone CH261-175O2 from chromosome z, complete sequence | 104 | 4.00E-18 | AC186343 | Z |
| Gallus gallus BAC clone CH261-55G9 from chromosome z, complete sequence | 104 | 4.00E-18 | AC186360 | Z |
| Gallus gallus BAC clone CH261-45C7 from chromosome w, complete sequence | 103 | 1.00E-17 | AC233977 | W |
| Gallus gallus BAC clone CH261-161O8 from chromosome z, complete sequence | 103 | 1.00E-17 | AC192618 | Z |
| Gallus gallus BAC clone CH261-144P8 from chromosome z, complete sequence | 103 | 1.00E-17 | AC192783 | Z |
| Gallus gallus BAC clone TAM31-62C15 from chromosome z, complete sequence | 103 | 1.00E-17 | AC188691 | Z |
| Gallus gallus BAC clone CH261-81D3 from chromosome w, complete sequence | 103 | 1.00E-17 | AC174867 | W |
| Gallus gallus BAC clone CH261-25P17 from chromosome z, complete sequence | 102 | 1.00E-17 | AC192757 | Z |
| Gallus gallus BAC clone CH261-78A20 from chromosome z, complete sequence | 102 | 1.00E-17 | AC204734 | Z |
| Gallus gallus BAC clone CH261-162G20 from chromosome w, complete sequence | 102 | 1.00E-17 | AC186171 | W |
| Gallus gallus BAC clone CH261-58O4 from chromosome z, complete sequence | 102 | 1.00E-17 | AC197155 | Z |
| Gallus gallus BAC clone CH261-90G19 from chromosome w, complete sequence | 102 | 1.00E-17 | AC205936 | W |
| Gallus gallus BAC clone CH261-132G22 from chromosome z, complete sequence | 102 | 1.00E-17 | AC188579 | Z |
| Gallus gallus BAC clone CH261-36M21 from chromosome z, complete sequence | 102 | 1.00E-17 | AC187998 | Z |
| Gallus gallus BAC clone TAM33-40B24 from chromosome ul, complete sequence | 102 | 1.00E-17 | AC140947 | 1 |
| Gallus gallus BAC clone CH261-94P4 from chromosome w, complete sequence | 101 | 5.00E-17 | AC239603 | W |
| Gallus gallus BAC clone CH261-65D3 from chromosome w, complete sequence | 101 | 5.00E-17 | AC239877 | W |
| Gallus gallus BAC clone CH261-115J22 from chromosome z, complete sequence | 101 | 5.00E-17 | AC186829 | Z |
| Gallus gallus BAC clone CH261-9F6 from chromosome z, complete sequence | 100 | 5.00E-17 | AC187117 | Z |
| Gallus gallus BAC clone CH261-112J14 from chromosome z, complete sequence | 100 | 5.00E-17 | AC187594 | Z |
| Gallus gallus fosmid J_AE-180G9, complete sequence | 99.6 | 2.00E-16 | AC270397 | W |
| Gallus gallus BAC clone CH261-9O22 from chromosome z, complete sequence | 99.6 | 2.00E-16 | AC202802 | Z |
| Gallus gallus BAC clone TAM31-48K11 from chromosome z, complete sequence | 99.6 | 2.00E-16 | AC192791 | Z |
| Gallus gallus BAC clone CH261-124L19 from chromosome z, complete sequence | 99.6 | 2.00E-16 | AC187592 | Z |
| Gallus gallus BAC clone TAM32-14C23 from chromosome z, complete sequence | 99.6 | 2.00E-16 | AC186872 | Z |
| Gallus gallus BAC clone CH261-118G10 from chromosome w, complete sequence | 98.7 | 2.00E-16 | AC239642 | W |
| Gallus gallus BAC clone CH261-110P23 from chromosome z, complete sequence | 98.7 | 2.00E-16 | AC187593 | Z |
| Gallus gallus BAC clone CH261-21K10 from chromosome z, complete sequence | 98.7 | 2.00E-16 | AC192060 | Z |
| Gallus gallus BAC clone CH261-171P12 from chromosome z, complete sequence | 98.7 | 2.00E-16 | AC190152 | Z |
| Gallus gallus BAC clone CH261-91O3 from chromosome z, complete sequence | 98.7 | 2.00E-16 | AC189111 | Z |
| Gallus gallus BAC clone CH261-138N19 from chromosome z, complete sequence | 97.8 | 6.00E-16 | AC211865 | Z |
| Gallus gallus BAC clone CH261-15G5 from chromosome z, complete sequence | 97.8 | 6.00E-16 | AC197813 | Z |
| Gallus gallus BAC clone CH261-137P21 from chromosome z, complete sequence | 97.8 | 6.00E-16 | AC186632 | Z |
| Gallus gallus BAC clone CH261-142P10 from chromosome z, complete sequence | 97.8 | 6.00E-16 | AC192782 | Z |
| Gallus gallus BAC clone CH261-100C1 from chromosome z, complete sequence | 97.8 | 6.00E-16 | AC191644 | Z |
| PREDICTED: Gallus gallus zinc finger protein 3-like (LOC107051198), mRNA | 96.9 | 6.00E-16 | XM_015274850 | 31 |
| PREDICTED: Gallus gallus zinc finger protein 239-like (LOC107049609), ncRNA | 96.9 | 6.00E-16 | XR_003072733 | 31 |
| Gallus gallus fosmid J_AD-672N11, complete sequence | 96.9 | 6.00E-16 | AC270379 | W |
| Gallus gallus fosmid J_AD-445J16, complete sequence | 96.9 | 6.00E-16 | AC270373 | W |
| Gallus gallus BAC clone CH261-107E4 from chromosome w, complete sequence | 96 | 2.00E-15 | AC239608 | W |
| Gallus gallus BAC clone CH261-32O1 from chromosome unknown, complete sequence | 96 | 2.00E-15 | AC145931 | 2 |
| Gallus gallus BAC clone TAM32-53B3 from chromosome z, complete sequence | 95.1 | 2.00E-15 | AC192558 | Z |
| Gallus gallus BAC clone CH261-113D21 from chromosome z, complete sequence | 94.2 | 8.00E-15 | AC189661 | Z |
| Gallus gallus BAC clone CH261-1G5 from chromosome z, complete sequence | 94.2 | 8.00E-15 | AC186859 | Z |
| Gallus gallus BAC clone CH261-9B3 from chromosome z, complete sequence | 94.2 | 8.00E-15 | AC186546 | Z |
| Gallus gallus BAC clone TAM33-8O11 from chromosome z, complete sequence | 94.2 | 8.00E-15 | AC186870 | Z |

**Table S2.** Genome content of green Anole (*Anolis carolinensis*) homologous to *Pogona vitticeps* BAC clone Pv151P16 as identified by BLASTn with the NCBI database (<http://blast.ncbi.nlm.nih.gov/Blast.cgi>); Aca: *Anolis carolinensis*.

| **Description** | **Score** | **E-value** | **Accession** | **Aca chromosomal location** |
| --- | --- | --- | --- | --- |
| PREDICTED: Anolis carolinensis zinc finger protein 271 (LOC100555897), mRNA | 949 | 0 | XM_008124874.2 | unknown |
| PREDICTED: Anolis carolinensis zinc finger protein 709-like (LOC107983865), partial mRNA | 948 | 0 | XM_016999221.1 | unknown |
| PREDICTED: Anolis carolinensis zinc finger protein 721-like (LOC103281413), mRNA | 1597 | 0 | XM_008122926.2 | unknown |
| PREDICTED: Anolis carolinensis zinc finger protein 709-like (LOC100562053), mRNA | 1324 | 0 | XM_016997927.1 | unknown |
| PREDICTED: Anolis carolinensis zinc finger protein 135-like (LOC107983708), mRNA | 1058 | 0 | XM_016998342.1 | unknown |
| PREDICTED: Anolis carolinensis zinc finger protein 135-like (LOC100566584), mRNA | 1040 | 0 | XM_016998432.1 | unknown |
| PREDICTED: Anolis carolinensis zinc finger protein 709 (LOC100551842), transcript variant X2, mRNA | 1039 | 0 | XM_016999049.1 | unknown |
| PREDICTED: Anolis carolinensis zinc finger protein 709 (LOC100551842), transcript variant X1, mRNA | 1039 | 0 | XM_008124429.2 | unknown |
| PREDICTED: Anolis carolinensis zinc finger protein 658B (LOC103281476), transcript variant X1, mRNA | 1599 | 0 | XM_016998398.1 | unknown |
| PREDICTED: Anolis carolinensis zinc finger protein 91-like (LOC100562188), mRNA | 1571 | 0 | XM_016998341.1 | unknown |
| PREDICTED: Anolis carolinensis zinc finger protein 91-like (LOC100562976), transcript variant X1, mRNA | 1565 | 0 | XM_016998078.1 | unknown |
| PREDICTED: Anolis carolinensis zinc finger protein 91-like (LOC100562976), transcript variant X2, mRNA | 1547 | 0 | XM_016998079.1 | unknown |
| PREDICTED: Anolis carolinensis zinc finger protein 239-like (LOC100558638), transcript variant X1, mRNA | 1526 | 0 | XM_016998880.1 | unknown |
| PREDICTED: Anolis carolinensis zinc finger protein 721 (LOC103281321), mRNA | 1503 | 0 | XM_008122655.2 | unknown |
| PREDICTED: Anolis carolinensis zinc finger protein 658B (LOC103281476), transcript variant X18, mRNA | 1483 | 0 | XM_016998415.1 | unknown |
| PREDICTED: Anolis carolinensis zinc finger protein 658B (LOC103281476), transcript variant X19, mRNA | 1480 | 0 | XM_016998416.1 | unknown |
| PREDICTED: Anolis carolinensis zinc finger protein 658B (LOC103281476), transcript variant X25, mRNA | 1436 | 0 | XM_016998422.1 | unknown |
| PREDICTED: Anolis carolinensis zinc finger protein 658B (LOC103281476), transcript variant X23, mRNA | 1430 | 0 | XM_016998420.1 | unknown |
| PREDICTED: Anolis carolinensis zinc finger protein 658B (LOC103281476), transcript variant X24, mRNA | 1423 | 0 | XM_016998421.1 | unknown |
| PREDICTED: Anolis carolinensis zinc finger protein 658B (LOC103281476), transcript variant X28, mRNA | 1412 | 0 | XM_016998425.1 | unknown |
| PREDICTED: Anolis carolinensis zinc finger protein 658B (LOC103281476), transcript variant X26, mRNA | 1400 | 0 | XM_016998423.1 | unknown |
| PREDICTED: Anolis carolinensis zinc finger protein 658B (LOC103281476), transcript variant X27, mRNA | 1391 | 0 | XM_016998424.1 | unknown |
| PREDICTED: Anolis carolinensis zinc finger protein 420-like (LOC100566973), transcript variant X1, mRNA | 1252 | 0 | XM_016998430.1 | unknown |
| PREDICTED: Anolis carolinensis zinc finger protein 658B (LOC103281476), transcript variant X30, mRNA | 1220 | 0 | XM_016998427.1 | unknown |
| PREDICTED: Anolis carolinensis zinc finger protein 658B (LOC103281476), transcript variant X31, mRNA | 1200 | 0 | XM_016998428.1 | unknown |
| PREDICTED: Anolis carolinensis zinc finger protein 709-like (LOC103278104), transcript variant X2, mRNA | 1081 | 0 | XM_016991898.1 | 2 |
| PREDICTED: Anolis carolinensis zinc finger protein 709-like (LOC103278104), transcript variant X1, mRNA | 1081 | 0 | XM_016991897.1 | 2 |
| PREDICTED: Anolis carolinensis zinc finger protein 709-like (LOC103277546), partial mRNA | 1074 | 0 | XM_016998847.1 | unknown |
| PREDICTED: Anolis carolinensis oocyte zinc finger protein XlCOF6 (LOC100557527), mRNA | 1041 | 0 | XM_016998067.1 | unknown |
| PREDICTED: Anolis carolinensis zinc finger protein 84-like (LOC107983719), transcript variant X2, mRNA | 1010 | 0 | XM_016998397.1 | unknown |
| PREDICTED: Anolis carolinensis zinc finger protein 709 (LOC103281127), transcript variant X2, mRNA | 979 | 0 | XM_008121976.2 | unknown |
| PREDICTED: Anolis carolinensis zinc finger protein 658B-like (LOC100560888), partial mRNA | 948 | 0 | XM_016998940.1 | unknown |
| PREDICTED: Anolis carolinensis zinc finger protein 721 (LOC100552818), mRNA | 1505 | 0 | XM_016998322.1 | unknown |
| PREDICTED: Anolis carolinensis zinc finger protein 658B (LOC103281476), transcript variant X15, mRNA | 1495 | 0 | XM_016998412.1 | unknown |
| PREDICTED: Anolis carolinensis zinc finger protein 658B (LOC103281476), transcript variant X16, mRNA | 1480 | 0 | XM_016998413.1 | unknown |
| PREDICTED: Anolis carolinensis zinc finger protein 658B (LOC103281476), transcript variant X12, mRNA | 1480 | 0 | XM_016998409.1 | unknown |
| PREDICTED: Anolis carolinensis zinc finger protein 658B (LOC103281476), transcript variant X11, mRNA | 1474 | 0 | XM_016998408.1 | unknown |
| PREDICTED: Anolis carolinensis zinc finger protein 658B (LOC103281476), transcript variant X13, mRNA | 1472 | 0 | XM_016998410.1 | unknown |
| PREDICTED: Anolis carolinensis zinc finger protein 658B (LOC103281476), transcript variant X8, mRNA | 1472 | 0 | XM_016998405.1 | unknown |
| PREDICTED: Anolis carolinensis zinc finger protein 658B (LOC103281476), transcript variant X14, mRNA | 1468 | 0 | XM_016998411.1 | unknown |
| PREDICTED: Anolis carolinensis zinc finger protein 658B (LOC103281476), transcript variant X9, mRNA | 1466 | 0 | XM_016998406.1 | unknown |
| PREDICTED: Anolis carolinensis zinc finger protein 658B (LOC103281476), transcript variant X22, mRNA | 1464 | 0 | XM_016998419.1 | unknown |
| PREDICTED: Anolis carolinensis zinc finger protein 658B (LOC103281476), transcript variant X10, mRNA | 1463 | 0 | XM_016998407.1 | unknown |
| PREDICTED: Anolis carolinensis zinc finger protein 658B (LOC103281476), transcript variant X6, mRNA | 1461 | 0 | XM_016998403.1 | unknown |
| PREDICTED: Anolis carolinensis zinc finger protein 658B (LOC103281476), transcript variant X21, mRNA | 1453 | 0 | XM_016998418.1 | unknown |
| PREDICTED: Anolis carolinensis zinc finger protein 658B (LOC103281476), transcript variant X17, mRNA | 1452 | 0 | XM_016998414.1 | unknown |
| PREDICTED: Anolis carolinensis zinc finger protein 658B (LOC103281476), transcript variant X2, mRNA | 1449 | 0 | XM_016998399.1 | unknown |
| PREDICTED: Anolis carolinensis zinc finger protein 658B (LOC103281476), transcript variant X20, mRNA | 1446 | 0 | XM_016998417.1 | unknown |
| PREDICTED: Anolis carolinensis zinc finger protein 658B (LOC103281476), transcript variant X3, mRNA | 1445 | 0 | XM_016998400.1 | unknown |
| PREDICTED: Anolis carolinensis zinc finger protein 658B (LOC103281476), transcript variant X7, mRNA | 1429 | 0 | XM_016998404.1 | unknown |
| PREDICTED: Anolis carolinensis zinc finger protein 658B (LOC103281476), transcript variant X5, mRNA | 1426 | 0 | XM_016998402.1 | unknown |
| PREDICTED: Anolis carolinensis zinc finger protein 658B (LOC103281476), transcript variant X4, mRNA | 1415 | 0 | XM_016998401.1 | unknown |
| PREDICTED: Anolis carolinensis zinc finger protein 850 (LOC103281604), transcript variant X3, mRNA | 1410 | 0 | XM_016998627.1 | unknown |
| PREDICTED: Anolis carolinensis zinc finger protein 850 (LOC103281604), transcript variant X1, mRNA | 1381 | 0 | XM_016998625.1 | unknown |
| PREDICTED: Anolis carolinensis oocyte zinc finger protein XlCOF6-like (LOC103281201), mRNA | 1378 | 0 | XM_016998068.1 | unknown |
| PREDICTED: Anolis carolinensis zinc finger protein 658B (LOC103281476), transcript variant X29, mRNA | 1321 | 0 | XM_016998426.1 | unknown |
| PREDICTED: Anolis carolinensis zinc finger protein 709-like (LOC103282061), mRNA | 1204 | 0 | XM_016999115.1 | unknown |
| PREDICTED: Anolis carolinensis zinc finger protein 420-like (LOC103281686), transcript variant X3, mRNA | 1137 | 0 | XM_008123749.2 | unknown |
| PREDICTED: Anolis carolinensis zinc finger protein 420-like (LOC103281686), transcript variant X2, mRNA | 1137 | 0 | XM_008123748.2 | unknown |
| PREDICTED: Anolis carolinensis zinc finger protein 420-like (LOC103281686), transcript variant X1, mRNA | 1137 | 0 | XM_008123747.2 | unknown |
| PREDICTED: Anolis carolinensis zinc finger protein 709 (LOC103281127), transcript variant X1, mRNA | 1123 | 0 | XM_016997928.1 | unknown |
| PREDICTED: Anolis carolinensis zinc finger protein 2 homolog (LOC100561859), mRNA | 1105 | 0 | XM_008121983.2 | unknown |
| PREDICTED: Anolis carolinensis zinc finger protein 658B-like (LOC103282590), mRNA | 1092 | 0 | XM_016999327.1 | unknown |
| PREDICTED: Anolis carolinensis zinc finger protein 420-like (LOC107983731), mRNA | 979 | 0 | XM_016998559.1 | unknown |
| PREDICTED: Anolis carolinensis zinc finger protein 420-like (LOC107983841), partial mRNA | 949 | 0 | XM_016999145.1 | unknown |
| PREDICTED: Anolis carolinensis zinc finger protein 470-like (LOC107982282), mRNA | 947 | 0 | XM_016998685.1 | unknown |
| PREDICTED: Anolis carolinensis zinc finger protein 850 (LOC103281604), transcript variant X4, mRNA | 1396 | 0 | XM_016998628.1 | unknown |
| PREDICTED: Anolis carolinensis zinc finger protein 850 (LOC103281604), transcript variant X2, mRNA | 1375 | 0 | XM_016998626.1 | unknown |
| PREDICTED: Anolis carolinensis zinc finger protein 850 (LOC103281604), transcript variant X5, mRNA | 1336 | 0 | XM_016998629.1 | unknown |
| PREDICTED: Anolis carolinensis zinc finger protein 91-like (LOC100562976), transcript variant X4, mRNA | 1286 | 0 | XM_016998081.1 | unknown |
| PREDICTED: Anolis carolinensis zinc finger protein 91-like (LOC100562976), transcript variant X3, mRNA | 1286 | 0 | XM_016998080.1 | unknown |
| PREDICTED: Anolis carolinensis zinc finger protein 850 (LOC103281604), transcript variant X6, mRNA | 1249 | 0 | XM_016998630.1 | unknown |
| PREDICTED: Anolis carolinensis oocyte zinc finger protein XlCOF6 (LOC100561863), mRNA | 1223 | 0 | XM_016998944.1 | unknown |
| PREDICTED: Anolis carolinensis zinc finger protein 850 (LOC103281604), transcript variant X7, mRNA | 1190 | 0 | XM_016998631.1 | unknown |
| PREDICTED: Anolis carolinensis zinc finger protein 850-like (LOC100557984), mRNA | 1043 | 0 | XM_016998110.1 | unknown |
| PREDICTED: Anolis carolinensis zinc finger protein 658B (LOC100552227), mRNA | 1041 | 0 | XM_003228541.3 | unknown |
| PREDICTED: Anolis carolinensis zinc finger protein 850 (LOC100568073), mRNA | 1035 | 0 | XM_016997755.1 | unknown |
| PREDICTED: Anolis carolinensis zinc finger protein 14 (znf14), partial mRNA | 1023 | 0 | XM_016998995.1 | unknown |
| PREDICTED: Anolis carolinensis gastrula zinc finger protein XlCGF26.1-like (LOC100559098), mRNA | 985 | 0 | XM_008122191.2 | unknown |
| PREDICTED: Anolis carolinensis zinc finger protein 420-like (LOC100564755), transcript variant X2, mRNA | 980 | 0 | XM_016998637.1 | unknown |
| PREDICTED: Anolis carolinensis oocyte zinc finger protein XlCOF6-like (LOC100558833), mRNA | 957 | 0 | XM_016999071.1 | unknown |
| Anolis carolinensis clone AnolisBov-B-LINE-140 Bov-B LINE sequence | 955 | 0 | FJ158982.1 | unknown |
| PREDICTED: Anolis carolinensis zinc finger protein 850 (LOC100567107), mRNA | 1219 | 0 | XM_003229602.3 | unknown |
| PREDICTED: Anolis carolinensis zinc finger protein 135-like (LOC100568271), transcript variant X3, mRNA | 1191 | 0 | XM_016997753.1 | unknown |
| PREDICTED: Anolis carolinensis zinc finger protein 850 (LOC103281405), mRNA | 1177 | 0 | XM_016998306.1 | unknown |
| PREDICTED: Anolis carolinensis zinc finger protein 850 (LOC100558637), mRNA | 1116 | 0 | XM_008123922.2 | unknown |
| PREDICTED: Anolis carolinensis zinc finger protein 850-like (LOC103282273), transcript variant X3, mRNA | 982 | 0 | XM_016999208.1 | unknown |
| PREDICTED: Anolis carolinensis zinc finger protein 850-like (LOC107983644), mRNA | 980 | 0 | XM_016998065.1 | unknown |
| PREDICTED: Anolis carolinensis zinc finger protein 850-like (LOC100555441), mRNA | 976 | 0 | XM_003230230.3 | unknown |
| PREDICTED: Anolis carolinensis zinc finger protein 850-like (LOC103282273), transcript variant X2, mRNA | 973 | 0 | XM_016999207.1 | unknown |
| PREDICTED: Anolis carolinensis zinc finger protein 850-like (LOC103282273), transcript variant X4, mRNA | 966 | 0 | XM_016999209.1 | unknown |
| PREDICTED: Anolis carolinensis zinc finger protein 721-like (LOC100558180), transcript variant X2, mRNA | 964 | 0 | XM_016998112.1 | unknown |
| PREDICTED: Anolis carolinensis zinc finger protein 721-like (LOC100558180), transcript variant X1, mRNA | 964 | 0 | XM_016998111.1 | unknown |
| PREDICTED: Anolis carolinensis zinc finger protein 850-like (LOC103282273), transcript variant X5, mRNA | 950 | 0 | XM_016999210.1 | unknown |
| PREDICTED: Anolis carolinensis zinc finger protein 850-like (LOC103282273), transcript variant X6, mRNA | 942 | 0 | XM_016999211.1 | unknown |
| PREDICTED: Anolis carolinensis zinc finger protein 135-like (LOC100568271), transcript variant X6, mRNA | 1179 | 0 | XM_016997754.1 | unknown |
| PREDICTED: Anolis carolinensis zinc finger protein 420-like (LOC100564755), transcript variant X1, mRNA | 1157 | 0 | XM_016998636.1 | unknown |
| PREDICTED: Anolis carolinensis zinc finger protein 135-like (LOC100568271), transcript variant X2, mRNA | 1136 | 0 | XM_016997752.1 | unknown |
| PREDICTED: Anolis carolinensis zinc finger protein 135-like (LOC100568271), transcript variant X1, mRNA | 1114 | 0 | XM_016997751.1 | unknown |
| PREDICTED: Anolis carolinensis zinc finger protein 850-like (LOC103282273), transcript variant X1, mRNA | 1014 | 0 | XM_016999206.1 | unknown |
