# Supplementary figures and images for: ZW Sex Chromosomes in Australian Dragon Lizards (Agamidae) Originated from a Combination of Duplication and Translocation in the Nucleolar Organising Region
Source: Genes (Basel). 2019 Oct 30;10(11):861. doi: 10.3390/genes10110861 (PMC6895791; doi:10.3390/genes10110861)

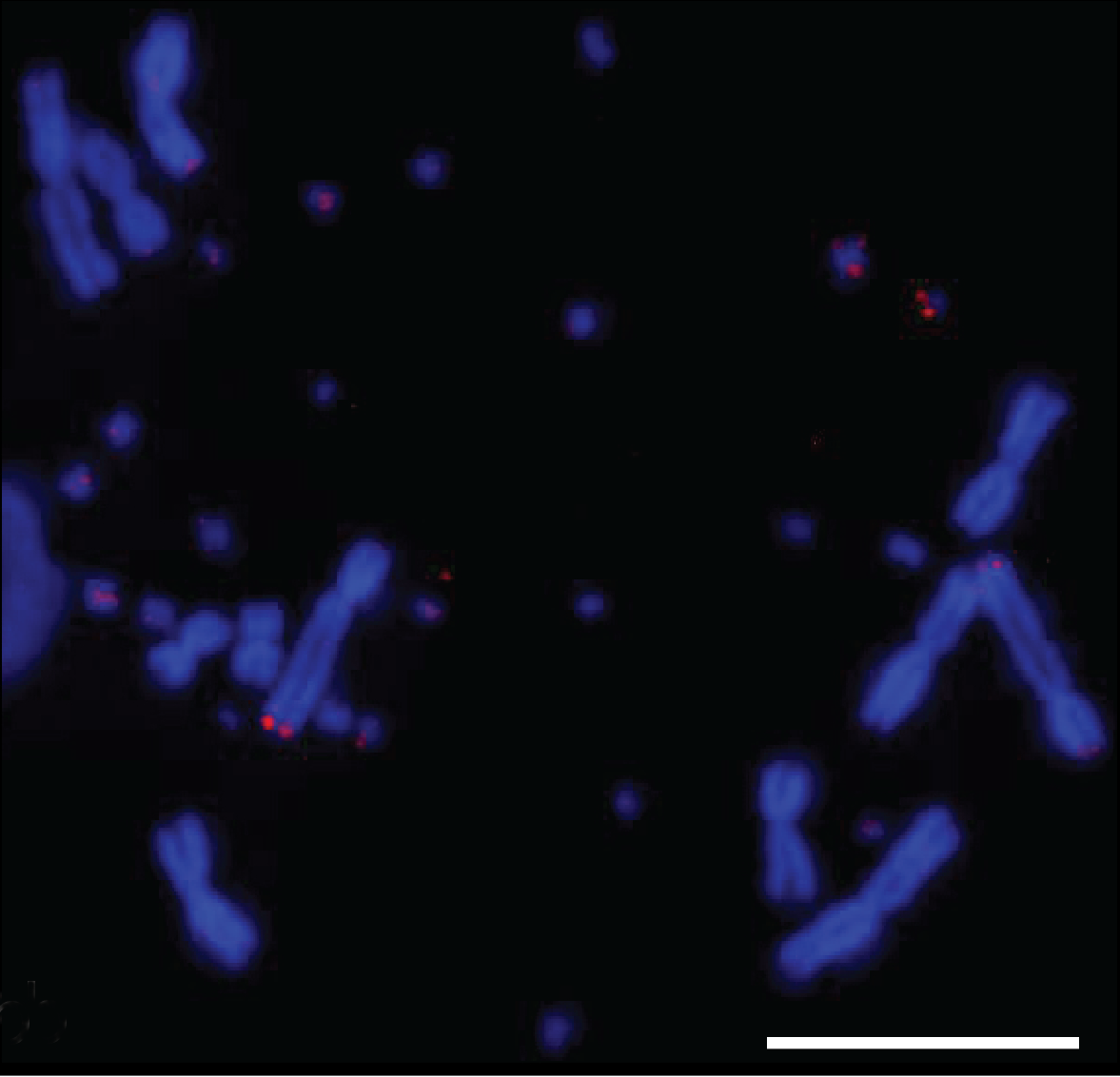

Supplement: Supplementary file 1 [file genes-10-00861-s001.zip › Genes_Matsubara_et_al_Figure S1.png]
